# Estimation of prediction error variances via Monte Carlo sampling methods using different formulations of the prediction error variance

**DOI:** 10.1186/1297-9686-41-23

**Published:** 2009-02-09

**Authors:** John M Hickey, Roel F Veerkamp, Mario PL Calus, Han A Mulder, Robin Thompson

**Affiliations:** 1 Animal Breeding and Genomics Centre, Animal Sciences Group, PO Box 65, 8200 AB, Lelystad, The Netherlands; 2 Grange Beef Research Centre, Teagasc, Dunsany, Co. Meath, Ireland; 3 School of Agriculture, Food and Veterinary Medicine, College of Life Sciences, University College Dublin, Belfield, Dublin 4, Ireland; 4 School of Mathematical Sciences, Queen Mary, University of London, Mile End Road, London E1 4NS, UK; 5 Centre for Mathematical and Computational Biology, Rothamsted Research, Harpenden AL5 2JQ, UK; 6 Department of Biomathematics and Bioinformatics, Rothamsted Research, Harpenden AL5 2JQ, UK

## Abstract

Calculation of the exact prediction error variance covariance matrix is often computationally too demanding, which limits its application in REML algorithms, the calculation of accuracies of estimated breeding values and the control of variance of response to selection. Alternatively Monte Carlo sampling can be used to calculate approximations of the prediction error variance, which converge to the true values if enough samples are used. However, in practical situations the number of samples, which are computationally feasible, is limited. The objective of this study was to compare the convergence rate of different formulations of the prediction error variance calculated using Monte Carlo sampling. Four of these formulations were published, four were corresponding alternative versions, and two were derived as part of this study. The different formulations had different convergence rates and these were shown to depend on the number of samples and on the level of prediction error variance. Four formulations were competitive and these made use of information on either the variance of the estimated breeding value and on the variance of the true breeding value minus the estimated breeding value or on the covariance between the true and estimated breeding values.

## Introduction

In quantitative genetics the prediction error variance-covariance matrix is central to the calculation of accuracies of estimated breeding values ($\hat{u}$) [*e.g. *[1]], to REML algorithms for the estimation of variance components [2], to methods which restrict the variance of response to selection [3], and can be used to explore trends in Mendelian sampling deviations over time [4]. The mixed model equations (**MME**) for most national genetic evaluations range from 100,000 to 20,000,000 equations and inversion of systems of equations of this size is generally not possible because of their magnitude or because of loss of numerical precision [5]. Methods that approximate the prediction error variances (**PEV**) and calculate the accuracy of $\hat{\text{u}}$ provide biased estimates in some circumstances by ignoring certain information [*e.g. *[6]]. Variance components upon which genetic evaluations of large populations are based are generally estimated using reduced data sets. The use of reduced data sets may create bias in the estimates as REML only provides unbiased estimates of variance components when all the data on which selection has taken place is included in the analysis [7]. Variance of response to selection is generally not controlled in breeding programs although it might be a risk to them [3].

Approximations of the PEV without needing to invert the coefficient matrix or to delete data, can be obtained by comparing Monte Carlo samples of the data and successive solutions of the mixed model equations of this data.

However different formulations have been presented to approximate the PEV in this way [8-11]. Approximations of the PEV using these formulations converge to the exact PEV (**PEV**_**exact**_) as the number of Monte Carlo samples increases, but the number of samples is generally limited by computational requirements in practice [*e.g. *[12]]. Also, differences in the rates of convergence have been shown to depend on the level of PEV_exact _for a given genetic variance ($\sigma_{g}^{2}$) [10]. Consequently, when finding the optimal number of iterations required, both the different formulations, and the level of PEV_exact _need to be taken into account. Some of the formulations are weighted averages of other formulations, with the weighting depending on the sampling variances of these. Garcia-Cortes *et al. *[10] use asymptotic approximations of these sampling variances. Alternative weighting strategies could use empirically approximated sampling variances based on independent replicates of samples or using leave-one-out Jackknife procedures [13,14].

The objective of this study was to compare the convergence to PEV_exact _of ten different formulations of the PEV, using simulations based on data and pedigree from a commercial population containing animals with different levels of PEV and using different numbers of samples (*n *= 50, 100, ..., 950, 1000). Four of the formulations were previously published, four were alternative versions of these, and two were derived as part of this study.

## Methods

### Monte Carlo sampling procedure for calculating PEV

The Monte Carlo sampling procedure for calculating the sampled PEV has been described extensively elsewhere for single breed [8-10] and multiple breed scenarios [12]. Assuming a simple additive genetic animal model without genetic groups **y **= **Xb **+ **Zu **+ **e**, where the distribution of random variables is **y **~ ***N***(**Xb**, **ZGZ**' + **R**), **u **~ ***N***(**0**, **G**), and **e **~ ***N***(**0**, **R**), the three steps involved in calculating the sampled PEV are as follows: **1. **Simulate *n *samples of **y **and **u **using the pedigree and the distributions of the original data, modified to account for the fact that the expectation of **Xb **does not affect the distribution of random variables [15,16] thus the samples of **y **can be simulated using random normal deviates from ***N***(**0**, **ZGZ**' + **R**) instead of ***N***(**Xb**, **ZGZ**' + **R**). **2. **Set up and solve the mixed model equations for the data set using the *n *simulated samples of **y **instead of the true **y**. This accounts for the fixed effects structure of the real data. **3. **Calculate the sampled PEV for some formulation.

### Formulations of PEV

Ten formulations of the sampled PEV are shown in Table 1. The first three formulations (PEV_GC1_, PEV_GC2_, and PEV_GC3_) were outlined by Garcia-Cortes *et al. *[10] and the fourth formulation (PEV_FL_) was outlined by Fouilloux and Laloë [8]. PEV_AF1_, PEV_AF2_, PEV_AF3_, and PEV_AF4 _are alternative versions of these formulations, which rescale the formulations from the Var (u) and to the $\sigma_{g}^{2}$ in order to account for the effects of sampling on the Var(u). Two new formulations of the sampled PEV (PEV_NF1_, and PEV_NF2_) are also given in Table 1. The ten formulations differ from each other in the way in which they compare information relating to the Var(u), the Var($\hat{\text{u}}$), the Var (u - $\hat{\text{u}}$), or the Cov(u, $\hat{\text{u}}$).

**Table 1:** Previously published, alternative, and new formulations of the prediction error variance for a random effect *u *with $\sigma_{g}^{2}$, the assumptions pertinent to each formulation, the information used in each formulation, and the asymptotic sampling variances of each formulation

Formulation	Assumptions	Uses information on	Asymptotic sampling variance
^1^PEV_GC1 _= $\sigma_{\text{g}}^{\text{2}}$ - Var($\hat{\text{u}}$)	Cov(u, $\hat{\text{u}}$) = Var($\hat{\text{u}}$) Var(u) = $\sigma_{\text{g}}^{\text{2}}$	$\hat{\text{u}}$	2*r*^4 ^$\sigma_{\text{g}}^{4}$/*n*
^2^PEV_GC2 _= Var(u - $\hat{\text{u}}$)	^11^Cov(u, $\hat{\text{u}}$) ≠/= Var($\hat{\text{u}}$) Var(u) = $\sigma_{\text{g}}^{\text{2}}$	u - $\hat{\text{u}}$	2(1-*r*^2^)^2 ^$\sigma_{\text{g}}^{4}$/*n*
^3 ^${\text{PEV}}_{\text{GC3}}=\frac{\left[\frac{{\text{PEV}}_{\text{GC1}}}{{\text{Var(PEV}}_{\text{GC1}}\text{)}}\right]+\left[\frac{{\text{PEV}}_{\text{GC2}}}{{\text{Var(PEV}}_{\text{GC2}}\text{)}}\right]}{\frac{\text{1}}{{\text{Var(PEV}}_{\text{GC1}}\text{)}}+\frac{\text{1}}{{\text{Var(PEV}}_{\text{GC2}}\text{)}}}$	Cov(u - $\hat{\text{u}}$, $\hat{\text{u}}$) = 0 Var(u) = $\sigma_{\text{g}}^{\text{2}}$	$\hat{\text{u}}$, u - $\hat{\text{u}}$	{[2*r*^4^(1-*r*^2^)^2^]/[(1-*r*^2^)^2 ^+ *r*^4^]}$\sigma_{\text{g}}^{4}$/*n*
^4^PEV_FL _= $\sigma_{\text{g}}^{\text{2}}$ - Cov(u, $\hat{\text{u}}$)	Cov(u, $\hat{\text{u}}$) = Var($\hat{\text{u}}$) Var(u) = $\sigma_{\text{g}}^{\text{2}}$	Cov(u, $\hat{\text{u}}$)	*r*^2^(1+*r*^2^)$\sigma_{g}^{2}$/*n*
^5^PEV_AF1 _= $\sigma_{\text{g}}^{\text{2}}$ - [Var($\hat{\text{u}}$)/Var(u)] $\sigma_{\text{g}}^{\text{2}}$	Cov(u, $\hat{\text{u}}$) = Var($\hat{\text{u}}$) Var(u) ≠ $\sigma_{\text{g}}^{\text{2}}$	$\hat{\text{u}}$, u	4*r*^4^(1-*r*^2^)$\sigma_{\text{g}}^{4}$/*n*
^6^PEV_AF2 _= [Var(u - $\hat{\text{u}}$)/Var(u)] $\sigma_{\text{g}}^{\text{2}}$	^11^Cov(u, $\hat{\text{u}}$) ≠/= Var($\hat{\text{u}}$) Var(u) ≠ $\sigma_{\text{g}}^{\text{2}}$	u - $\hat{\text{u}}$, u	4*r*^2^(1-*r*^2^)^2 ^$\sigma_{\text{g}}^{4}$/*n*
^7 ^${\text{PEV}}_{\text{AF3}}=\frac{\left[\frac{{\text{PEV}}_{\text{AF1}}}{{\text{Var(PEV}}_{\text{AF1}}\text{)}}\right]+\left[\frac{{\text{PEV}}_{\text{AF2}}}{{\text{Var(PEV}}_{\text{AF2}}\text{)}}\right]}{\frac{\text{1}}{{\text{Var(PEV}}_{\text{AF1}}\text{)}}+\frac{\text{1}}{{\text{Var(PEV}}_{\text{AF2}}\text{)}}}$	Cov(u - $\hat{\text{u}}$, $\hat{\text{u}}$) = 0 Var(u) ≠ $\sigma_{\text{g}}^{\text{2}}$	$\hat{\text{u}}$, u - $\hat{\text{u}}$, u	4*r*^4 ^(1 - *r*^2^)^2 ^$\sigma_{\text{g}}^{4}$/*n*
^8^PEV_AF4 _= $\sigma_{\text{g}}^{\text{2}}$ - [Cov(u, $\hat{\text{u}}$)/Var(u)] $\sigma_{\text{g}}^{\text{2}}$	Cov(u, $\hat{\text{u}}$) = Var($\hat{\text{u}}$) Var(u) ≠ $\sigma_{\text{g}}^{\text{2}}$	Cov(u, $\hat{\text{u}}$), u	*r*^2^(1-*r*^2^)$\sigma_{g}^{2}$/*n*
^9^PEV_NF1 _= [1 - Cov(u, $\hat{\text{u}}$)^2^/(Var(u) × Var($\hat{\text{u}}$))]$\sigma_{\text{g}}^{\text{2}}$			4*r*^2^(1-*r*^2^)^2 ^$\sigma_{g}^{2}$/*n*
^10^PEV_NF2 _= {Var(u - $\hat{\text{u}}$)/[Var($\hat{\text{u}}$) + Var(u - $\hat{\text{u}}$]}$\sigma_{\text{g}}^{\text{2}}$	Cov(u - $\hat{\text{u}}$, $\hat{\text{u}}$) = 0	$\hat{\text{u}}$ and u - $\hat{\text{u}}$	4*r*^4^(1-*r*^2^)^2 ^$\sigma_{\text{g}}^{4}$/*n*

^1^Garcia-Cortes *et al*. (1995) formulation 1

^2^Garcia-Cortes *et al*. (1995) formulation 2

^3^Garcia-Cortes *et al*. (1995) formulation 3

^4^Fouilloux and Laloë (2001) formulation

^5^Corrects PEV_GC1 _for Var(u) ≠ $\sigma_{\text{g}}^{\text{2}}$ and corresponds to Lidauer *et al*. (2007)

^6^Corrects PEV_GC2 _for Var(u) ≠ $\sigma_{\text{g}}^{\text{2}}$

^7^Corrects PEV_GC3 _for Var(u) ≠ $\sigma_{\text{g}}^{\text{2}}$

^8^Corrects PEV_FL _for Var(u) ≠ $\sigma_{\text{g}}^{\text{2}}$

^9^Based on the classical formulation of the accuracy of an EBV

^10^Implicitly weighs information on Var ($\hat{\text{u}}$) and Var(u, $\hat{\text{u}}$) and corrects for Var(u) ≠ $\sigma_{\text{g}}^{\text{2}}$

^11^No assumption made about the relationship between Var($\hat{\text{u}}$)and Cov(u, $\hat{\text{u}}$)

### Approximation of sampling variance of PEV

Formulae, based on Taylor series approximations, to predict the asymptotic sampling variances for each of the ten formulations of sampled PEV at different levels of PEV_exact _are given in Table 1. The sampling variance can also be approximated stochastically using a number (*e.g. *100) of independent replicates of the *n *samples or by applying a leave-one-out Jackknife [13,14] to the *n *samples.

### Application to test data set

#### Data and model

A data set containing 32,128 purebred Limousin animals with records for a trait (height) and a corresponding pedigree of 50,435 animals was extracted from the Irish Cattle Breeding Federation database. In the simulations the trait was assumed to have a $\sigma_{g}^{2}$ of 1.0 and residual variance $\sigma_{r}^{2}$ of 3.0. Fixed effects were contemporary group, technician who scored the animal, parity of dam, age of animal at scoring and sex.

#### Calculation of exact PEV

The PEV_exact _were calculated for the extracted data set by setting up and solving the MME, with fixed effects of contemporary group, technician who scored the animal, parity of dam, and a second order polynomial of age of animal at scoring nested within sex, and random animal and residual effects, using the BLUP option in ASReml [17] which fully inverts the left hand side of the MME.

#### Sampled PEV

Following the Monte Carlo sampling procedure described above, 100,000 samples of the extracted data set were simulated assuming a $\sigma_{g}^{2}$ of 1.0 and $\sigma_{r}^{2}$ of 3.0. For each of the simulated data sets MME, using the same design matrix (X) as used when estimating the PEV_exact_, were set up and solved using MiX99 [18]. The sampled PEV of the $\hat{\text{u}}$ for each animal in the pedigree was approximated using the formulations of the sampled PEV described in Table 1 using *n *samples (*n *= 50, 100, ..., 950, 1000).

Stochastic approximations of the sampling variance of the sampled PEV were calculated using 100 independent replicates of the *n *samples, and using the leave-one-out Jackknife on *n *samples, for the different formulations, with the exception of PEV_GC3 _and PEV_AF3_. To calculate the sampling variance for PEV_GC3 _and PEV_AF3 _using *n *independent replicates would have required more than 100,000 samples (due to the need to generate sampling variances of component formulations) generated for this study so therefore these were not considered. Asymptotic sampling variances for all ten formulations were calculated using the formulae in Table 1.

### Alternative weighting strategies

Of the formulations presented in Table 1, PEV_GC3 _and PEV_AF3 _are weighted averages of PEV_GC1 _and PEV_GC2 _and of PEV_AF1 _and PEV_AF2 _respectively with the weighting dependent on the sampling variances of the component formulations. Garcia-Cortes *et al. *[10] suggest weighting by asymptotic approximations of the sampling variances. The sampling variances could also be approximated empirically using independent replicates of *n *samples or by leave-one-out Jackknife procedures [13,14]. These alternative weighting strategies were compared by calculating sampling variances using 100 independent replicates of the *n *samples, using the *n *samples and a leave-one-out Jackknife procedure [14], and using the asymptotic sampling variances outlined in Table 1 as part of an iterative procedure, which involved two iterations. In the first iterations the asymptotic sampling variances were calculated using the PEV_GC1 _and PEV_GC2 _of the component formulations, in the second they used the PEV_GC3 _approximated in the first iteration.

### Calculation of required variances and covariances

It was not possible to store each of the 100,000 simulated values for each of the 50,435 animals in the main memory of the computer simultaneously meaning that textbook formulae to calculate the different variances and covariances required for the different formulations was not possible. Textbook updating algorithms to calculate the variance can be numerically unreliable [19]. Instead the required variances were calculated using a one pass updating algorithm based on Chan *et al. *[19] which updates the estimated sum of squares with a new record as it reads through the data and takes the form:

Sn=Sn−1+((n−1)([(Tn−1n−1)−xi]2n)),

where *n *are the number samples at any stage in the updating procedure and *T *and *S *are the sum and sum of squares of the data points 1 through *n*. It was modified to calculate the covariances between X and Y by changing [(Tn−1n−1)−xi]2 to [(Txn−1n−1)−xi]×[(Tyn−1n−1)−yi]. Both of these algorithms were tested using one replication of 100,000 samples and found to be stable.

## Results

As the $\sigma_{g}^{2}$ was taken to be 1.0, the PEV ranged between 0.00 and 1.0. For the purpose of categorizing the results PEV with values between 0.00 and 0.33 were regarded as low, values between 0.34 and 0.66 were regarded as medium, and values between 0.67 and 1.00 were regarded as high.

Henderson [20] showed that it is much easier to form **A**^-1 ^than **A**, where **A **is the numerator relationship matrix among animals. This follows from the fact that, if the individuals are listed with ancestors above descendants, **A **can be written as **TMT**' where **M **is a diagonal matrix and **T **is a lower triangular matrix with non-zero diagonal elements and *i*, *j *th elements that are non-zero if the *j *th individual is an ancestor of the *i *th [21]. The matrix **T **has a simple inverse with both the diagonal elements and *i*, *j *th elements being non-zero if the *j *th individual is a parent of the *i *th individual. Hence **A **has a simple inverse. It is interesting to note that an animal effect can be written as an accumulation of independent terms from its ancestors $u_{i}=\frac{\left(u_{si}+u_{di}\right)}{2}+m_{i}$, where *u*_*si *_and *u*_*di *_are the additive genetic effects of the sire and dam of animal *i *and *m*_*i *_is the Mendelian sampling effect with variance $A_{m_{i}}=\frac{\left(1-F_{i}\right)}{2}\sigma_{g}^{2}$, where *F*_*i *_is the average inbreeding of the parents of animal *i*. Hence there is a simple recursive procedure for generation of the additive effects *u*_*i *_by generating independent Mendelian sampling terms *m*_*i *_with diagonal variance matrix $A_{m_{i}}$.

### General trends of sampled PEV

**Figure 1:**
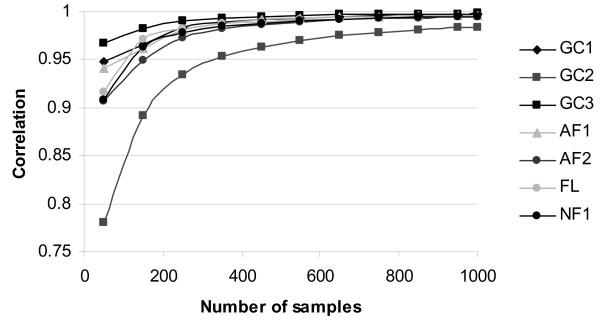
**Correlations between exact prediction error variance and different formulations of sampled prediction error variance^1 ^using *n *samples (*n *= 50, 100, ..., 950, 1000), for 18,855 non-inbred animals**. ^1^PEV_NF2_, PEV_AF3_, PEV_AF4 _are not shown as they have trends, which match PEV_GC3_

While all different formulations of the sampled PEV converged to the PEV_exact _and the sampling variance of the PEV reduced as the number of samples (*n*) increased, convergence rates differed between the formulations. For example, PEV_GC2 _converged at a slower rate than all other formulations when the convergence rate was measured by the correlation between PEV_exact _and sampled PEV (Fig. 1). PEV_GC1_, PEV_AF3_, PEV_AF4_, and PEV_NF2_, all converged at a very similar rates and had the best convergence across all formulations.

**Figure 2:**
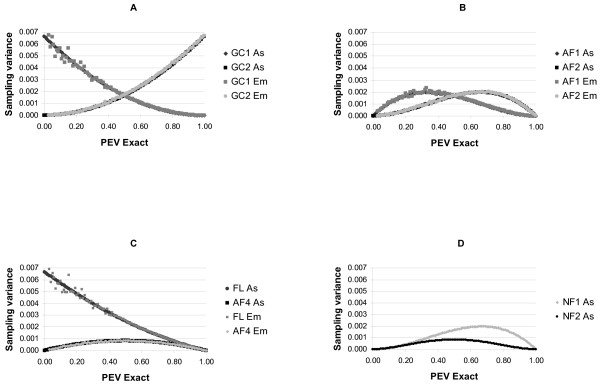
**Sampling variances of sampled prediction error variance approximated asymptotically (As) and empirically^1 ^(Em) using different formulations of the prediction error variance using 300 samples for different levels of exact prediction error variance**. (**A**) Sampling variances for PEV_GC1 _and PEV_GC2_. (**B**) Sampling variances for PEV_AF1 _and PEV_AF2_. (**C**) Sampling variances for PEV_FL _and PEV_AF4_. (**D**) Sampling variances for PEV_NF1 _and PEV_NF2_^2^. ^1^Empirical sampling variances were approximated using 100 independent replicates and presented as averages within windows of 0.001 of the exact prediction error variance. ^2^PEV_GC3_, and PEV_AF3 _were similar to PEV_NF2_.

As well as depending on the numbers of samples, the convergence rate also depended on the level of the PEV_exact_. The sampled PEV calculated using different formulations had different sampling variances and within each formulation the sampling variances differed depending on the level of the PEV_exact _(Fig. 2). Of the previously published formulations PEV_GC1 _and PEV_FL _had low sampling variance at high PEV_exact_, with PEV_GC1 _being better than PEV_FL_. PEV_GC2 _had low sampling variance at low PEV_exact_. Accounting for the effects of sampling on the Var(u) reduced the sampling variance in regions where the previously published formulations had high sampling variances but had little (or even slightly negative) effect where these formulations had low sampling variances. PEV_AF4_, which is the alternative version of PEV_FL _gave major improvements in terms of sampling variance low and intermediate PEV_exact_. Its performance was almost identical to PEV_NF2_, PEV_AF3_, and PEV_GC3_, which had low sampling variance at both high and low PEV. No formulation had relatively low sampling variance for intermediate PEV.

### Comparison of formulations

Different formulations were compared in greater detail using *n *= 300 samples (Table 2), which is a practical number of samples. PEV_GC3_, PEV_AF3_, PEV_AF4_, and PEV_NF2 _were the best formulations across all of the ten formulations. The slopes and R^2 ^of their regressions were always among the best where PEV_exact _was low, intermediate, or high (Table 2). These formulations gave good approximations at both high and low PEV_exact _their performance was less good at intermediate PEV, measured by each of the summary statistics (Table 2).

**Table 2:** Intercept, slope, R^2^, and root mean squared error (RMSE) of regressions of exact prediction error variance on sampled prediction error variance approximated using one of 10 different formulations of the prediction error variance using 300 samples, for 18,855 non-inbred animals

	PEV_exact_	PEV_GC1_	PEV_GC2_	PEV_GC3_	PEV_FL_	PEV_AF1_	PEV_AF2_	PEV_AF3_	PEV_AF4_	PEV_NF1_	PEV_NF2_
**Intercept**	0.00–0.33	0.09	0.01	0.01	0.09	0.05	0.02	0.01	0.02	0.01	0.01
	0.34–0.66	0.26	0.32	0.17	0.31	0.27	0.30	0.18	0.18	0.29	0.17
	0.67–1.00	0.09	0.29	0.06	0.05	0.09	0.06	0.02	0.02	0.04	0.04
											
**Slope**											
	0.00–0.33	0.62	0.90	0.93	0.62	0.77	0.89	0.93	0.93	0.91	0.95
	0.34–0.66	0.57	0.43	0.71	0.47	0.54	0.48	0.68	0.69	0.49	0.71
	0.67–1.00	0.91	0.67	0.94	0.95	0.91	0.93	0.98	0.97	0.96	0.96
											
**R**^2^	0.00–0.33	0.65	0.94	0.95	0.65	0.76	0.91	0.95	0.94	0.93	0.95
	0.34–0.66	0.59	0.43	0.68	0.49	0.54	0.48	0.67	0.69	0.49	0.70
	0.67–1.00	0.96	0.64	0.97	0.97	0.95	0.90	0.98	0.98	0.92	0.98
											
**RMSE**	0.00–0.33	0.05	0.02	0.02	0.05	0.04	0.03	0.02	0.02	0.02	0.02
	0.34–0.66	0.03	0.03	0.02	0.03	0.03	0.03	0.02	0.02	0.03	0.02
	0.67–1.00	0.02	0.06	0.02	0.02	0.02	0.03	0.01	0.02	0.03	0.01

PEV_GC1 _and PEV_FL _gave good approximations for high PEV_exact _and poor approximations for low PEV_exact_. PEV_GC2 _gave good approximations for low PEV_exact _and poor approximations for high PEV_exact_. Improving the published formulations by correcting for the effects of sampling resulted in better approximations in areas where the published formulations were weak. Slight (dis)improvements were observed where the previously published formulations were strong. Of the new formulations PEV_NF1 _gave poor approximations and PEV_NF2 _gave good approximations.

Using the three alternative weighting strategies to combine the component formulations for PEV_GC3 _and PEV_AF3 _gave almost identical results (Table 3).

**Table 3:** Coefficients of regressions of PEV_GC3_and PEV_AF3 _(sampling variances calculated empirically) on PEV_GC3 _and PEV_AF3 _(sampling variances calculated using Jackknife) and on PEV_GC3 _and PEV_AF3 _(sampling variances calculated asymptotically and weighting done iteratively)

	Jackknife		Asymptotic	
	PEV_GC3_	PEV_AF3_	PEV_GC3_	PEV_AF3_
**Intercept**	0.00	0.00	0.00	0.01
**Slope**	1.00	1.00	1.00	1.00
**R**^2^	1.00	1.00	1.00	1.00
**RMSE**	0.01	0.00	0.00	0.01

### Required number of samples

The formulations PEV_GC3_, PEV_AF3_, PEV_AF4_, and PEV_NF2 _gave similar approximations and had the lowest sampling variance. Even when a few samples (*n *= 50) were used, low and high PEV were well approximated and intermediate PEV_exact _were poorly approximated. Correlations between PEV_NF2 _and PEV_exact _were 0.88 for low, 0.96 for high PEV_exact _and 0.51 for intermediate PEV_exact_. To increase the correlation for intermediate PEV_exact _to at least 0.90 at least 550 samples was needed. At this number of samples the correlations for low and high PEV_exact _were ≥ 0.99. To obtain a satisfactory level of convergence 300 samples were sufficient.

## Discussion

### Differences between formulations

**Figure 3:**
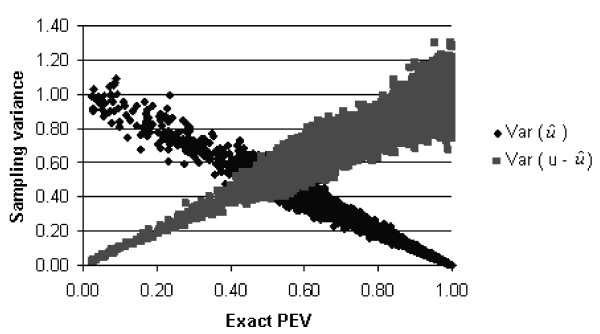
**X-Y plot of the exact prediction error variance and the Var($\hat{\text{u}}$) and Var(u - $\hat{\text{u}}$)**.

Ten different formulations of the PEV approximated using sampling were compared and these were each shown to converge to the PEV_exact _at different rates. Within each of these formulations differences in convergence were observed at different levels of PEV_exact_. PEV_GC1 _and its corresponding alternative formulation PEV_AF1 _make use of information on the Var($\hat{\text{u}}$). PEV_GC2 _and its corresponding alternative formulation PEV_AF2 _makes use of information on the Var(u - $\hat{\text{u}}$). The sampling variance of the Var($\hat{\text{u}}$) is lower at high PEV_exact _than it is at low PEV_exact _(Fig. 3), therefore the formulations using information on the Var($\hat{\text{u}}$) are more suited to approximating high PEV_exact _than to low PEV_exact_. The opposite is the case for formulations which use information on the Var(u - $\hat{\text{u}}$), they perform better at low PEV_exact_. Formulations PEV_GC3_, PEV_AF3_, and PEV_NF2 _use information on both the Var($\hat{\text{u}}$)and the Var (u - $\hat{\text{u}}$) and result in curves for their sampling variance which are symmetric about the mean PEV_exact_. They either explicitly or implicitly weight this information by the inverse of its sampling variance. PEV_FL _and PEV_AF4 _make use of information on the C*ov*(u, $\hat{\text{u}}$).

With infinite samples the Var(u) is equal to the $\sigma_{g}^{2}$, but due to sampling error resulting from using a limited number of samples this not likely to be true in practice. Therefore each of the alternative formulations makes use of information on the Var(u) in addition to making use of information on either/or/both of the Var($\hat{\text{u}}$) and the Var(u - $\hat{\text{u}}$) or the C*ov*(u, $\hat{\text{u}}$). The Var($\hat{\text{u}}$) = Cov(u, $\hat{\text{u}}$) when the Cov((u - $\hat{\text{u}}$), $\hat{\text{u}}$) = 0. The Var($\hat{\text{u}}$) ≠ Cov(u, $\hat{\text{u}}$) when the Cov((u - $\hat{\text{u}}$), $\hat{\text{u}}$) ≠ 0.

### Competitive formulations

Of the ten different approaches four competitive formulations, PEV_GC3_, PEV_AF3_, PEV_AF4_, and PEV_NF2_, were identified. These gave similar approximations. Of the four, two, PEV_GC3 _and PEV_AF3_, were weighted averages of component formulations. The weighting was based on the sampling variances of their component formulations. These sampling variances can be calculated using a number of independent replicates, using Jackknife procedures, or asymptotically. Each of these approaches gave almost identical results but the Jackknife and asymptotic approaches were far less computationally demanding.

### Computational time

A single BLUP evaluation for the routine Irish multiple breed beef genetic cattle evaluation (January 2007) which included a pedigree of 1,500,000 and 493,092 animals with performance records on at least one of the 15 traits could be run using MiX99 [18] in 366 min on a 64 bit PC, with a 2.40 GHz AMD Opteron dual-core processor and 8 gigabytes of RAM [12]. Using *n *= 300 samples and PEV_NF2 _the accuracy of the estimated breeding values could be estimated in 1,830 hours on a single processor. Several samples can be solved simultaneously on multiple processors thereby reducing computer time. Nowadays PC's are available that contain two quad core 64 bit processors (*i.e. *8 CPU's) and cost approximately 5,000 euro. Using six of these PC's the accuracy of estimated breeding values for the Irish data set could be estimated in less than 38.1 h.

### Application

The Monte Carlo sampling approach using one of these four competitive formulations can be used to improve many tasks in animal breeding. Stochastic REML algorithms [*e.g. *[9]] can be improved in terms of speed of calculation using these formulations, therefore allowing variance components to be estimated using REML in large data sets. These REML formulations are usually written in terms of additive genetic effects **u**'**A**^-1^**u **and *trace *[**A**^-1^**PEV**], where **PEV **is the prediction error covariance matrix for the estimated breeding values. The results of Henderson [22] show how the REML formulations can be equivalently written as in terms of Mendelian sampling effects *m ***m**'**A**^-1^**m **and *trace *[**A**_**m**_^-1^**PEV**_**m**_], where **PEV**_**m **_is the prediction error covariance matrix for the Mendelian sampling effects. As **A**_**m **_is diagonal we see that we only need to compute the sampling variances of the Mendelian sampling terms. When the sampling was carried out in this study we, in error, did not correct the Mendelian sampling terms for inbreeding. We therefore have only reported results for non-inbred animals and think that the incorrect generation will have a minimal effect on the sampling variances, which are presented as an empirical check on the formulae. There may be circumstances where a Stochastic REML approach may be faster than Gibbs sampling and have less bias than Method R [23]. Calculating variance components using more complete data sets would facilitate a reduction in the bias of estimated variance components caused by the ignoring of data on which selection has taken place in the population [12], due to computational limitations. Calculation of unbiased accuracy of within breed [8] and across breed [12] estimated breeding values can be improved by reducing the computational time required of calculation or reducing the sampling error for a given computational time. Application of an algorithm controlling the variance of response to selection [24] to large data sets can be speeded up. The variance of response to selection is a risk to breeding programs [3], which is generally not explicitly controlled using the approach outlined by Meuwissen [24] due to the inability to generate a prediction error (co)variance matrix for large data sets.

Computational power is a major limitation of stochastic methods, particularly when large data sets are involved, however this is dissipating rapidly with the improvement in processor speed, parallelization, and the adoption of 64-bit technology, however in the meantime deterministic methods will continue to be used for large scale BLUP analysis.

## Conclusion

PEV approximations using Monte Carlo estimation were affected by the formulation used to calculate the PEV. The difference between the formulations was small when the number of samples increased, but differed depending on the level of the exact PEV and the number of samples. Rescaling from the scale of Var(u) to the scale of $\sigma_{g}^{2}$ improved the approximation of the PEV and four of the 10 formulations gave the best approximations of PEV_exact _thereby improving the efficiency of the Monte Carlo sampling procedure for calculating the PEV. The fewer samples that are required the less the computational time will be.

## Competing interests

The authors declare that they have no competing interests.

## Authors' contributions

RT derived most of the mathematical equations. JH derived the remaining equations, carried out the simulations and wrote the first draft of the paper. RV supervised the research and mentored JH. MC and HM took part in useful discussions and advised on the simulations. All authors read and approved the final manuscript.
